# Association of regional specificity of blood-brain barrier permeability with pathological subtypes in cerebral white matter of patients with non-brain metastatic lung cancer

**DOI:** 10.3389/fonc.2026.1717244

**Published:** 2026-02-23

**Authors:** Haotian Wang, Jian Zeng, Zhengzhen Li, Yonglong Li, Yi Wang, Xiufu Zhang, Jun Zhou, Hongying Liu, Chunrong Wu, Ruipeng Liang

**Affiliations:** 1 Department of Radiology, Chongqing University Jiangjin Hospital, Chongqing, China; 2 Department of Oncology, Chongqing University Jiangjin Hospital, Chongqing, China

**Keywords:** blood-brain barrier, brain metastatic, cerebral white matter, DCE-MRI, lung cancer

## Abstract

**Objective:**

To investigate blood-brain barrier (BBB) permeability abnormalities in cerebral white matter regions of non-brain metastatic lung cancer (LC) patients and their association with histopathological subtypes.

**Methods:**

221 subjects (74 Healthy Controls [HC], 78 LC, 69 LC with Brain Metastasis [LCBM]) were enrolled. LC was subdivided into Adenocarcinoma (ADC), Squamous Cell Carcinoma (SCC), Small Cell Lung Cancer (SCLC). Dynamic Contrast-Enhanced MRI (DCE-MRI) assessed K^trans^, K_ep_, Ve, Vp in deep (frontal/parietal/temporal/occipital lobes) and periventricular (anterior/posterior horns) white matter. Non-parametric tests were used (p<0.05 significant).

**Results:**

LC group had K^trans^ comparable to LCBM but higher than HC (p<0.05), mainly in deep white matter and posterior periventricular regions (anterior periventricular: p>0.05). ADC showed higher K^trans^, K_ep_, Vp than SCC (all p<0.001) and SCLC (K^trans^/K_ep_ p<0.001; Vp p=0.71); Ve was lower than SCLC (p=0.003) but higher than SCC (p=0.001). SCLC had higher Ve than SCC (p<0.0001), with higher K^trans^/Vp than SCC (p<0.01). Vp correlated positively with K^trans^ in ADC/SCC (r=0.433/0.359, p<0.001), not in SCLC.

**Conclusion:**

LC impairs BBB integrity via paraneoplastic effects, with subtype heterogeneity: ADC (high perfusion/leakage) involves global white matter; SCLC (prominent interstitial disruption) affects mainly deep regions. Findings aid LC brain metastasis risk stratification and neuroprotective strategies.

## 1 Introduction

Lung cancer (LC), one of the malignant tumors with the highest incidence and mortality rates worldwide, exerts effects that extend far beyond local pulmonary lesions and gradually progress to systemic multisystem dysfunction (1, 2). Clinical studies have confirmed (3) that patients with non-brain metastatic LC may already present with neurological symptoms, such as cognitive dysfunction and cancer-related neuropathic pain, suggesting that LC exerts a “paraneoplastic effect” on the central nervous system. As a key structure for maintaining central nervous system homeostasis, the blood-brain barrier (BBB) may contribute to LC-related neurological complications through its structural and functional abnormalities, which are potentially core underlying mechanisms. However, existing studies (4, 5) have mostly focused on BBB changes in local brain metastases or macroscopic structural damage to the brain. As the primary region for housing nerve fiber tracts in the brain, abnormal BBB permeability in the cerebral white matter directly impairs nerve fiber signal transmission. Whether abnormal BBB permeability in the cerebral white matter of patients with non-brain metastatic LC is linked to the pathogenesis of neurological symptoms (e.g., cognitive impairment and cancer-related neuropathic pain) remains to be systematically investigated. Therefore, this study aims to explore the characteristics of abnormal BBB permeability in the cerebral white matter of patients with non-brain metastatic LC using dynamic contrast-enhanced magnetic resonance imaging (DCE-MRI), thereby providing an imaging-based theoretical basis for revealing the neuroimaging mechanism of the LC-related paraneoplastic effect, for developing brain metastasis risk stratification, and for formulating neuroprotective strategies.

A series of prospective cohort studies have confirmed that LC patients may exhibit cognitive abnormalities at the time of initial diagnosis (6–9), characterized by symptoms including inability to concentrate, impaired executive function, and memory decline; these abnormalities may further deteriorate following therapeutic interventions such as chemotherapy and radiotherapy. Studies have demonstrated that radiotherapy has been shown to exert region-specific effects on brain structure: patients with LC undergoing whole-brain radiotherapy exhibit significant hippocampal atrophy (a 5.7% volume reduction), while the volume of cerebral white matter decreases by only 1.3%—a rate not significantly different from that in patients treated with chemotherapy alone (10). This suggests that direct radiotherapy-induced damage to the cerebral white matter may be limited. In contrast, microenvironmental alterations in the cerebral white matter (e.g., abnormal BBB) mediated by the paraneoplastic effects of lung cancer may serve as an earlier pathological driver of cognitive impairment. Beyond cognitive dysfunction, cancer pain—one of the most common comorbidities in lung cancer patients (11–13)—has become a research focus due to its profound impacts on brain functional networks. Functional magnetic resonance imaging (fMRI) studies have revealed that LC-related cancer pain persistently activates the brain’s pain-processing network, inducing abnormal changes in functional connectivity strength within this network. Additionally, long-term cancer pain may disrupt the function of both the emotional regulation network and the reward network, which further exacerbates patients’ negative emotions (e.g., anxiety and depression) and contributes to the formation of a “pain-emotion-cognition” vicious cycle (14, 15). However, existing studies have primarily focused on functional and structural changes in gray matter regions (e.g., the cortex and nuclei), with relatively insufficient attention paid to white matter regions. As the primary region housing the brain’s nerve fiber tracts, white matter integrity directly dictates the efficiency of information transmission between distinct brain regions, and its structural and functional abnormalities may represent potential pathological mechanisms underlying LC-related cognitive impairment and pain network dysfunctions (13). Related studies using texture analysis have identified pre-existing microstructural changes in the bilateral frontoparietal white matter of LC patients without brain metastasis, which correlate with tumor stage and histopathological malignancy (16). This finding suggests that LC may damage the cerebral white matter via a paraneoplastic effect prior to brain metastasis, providing imaging evidence for early white matter injury in LC patients before the development of brain metastases. Further research has found that the topological properties of the cerebral white matter structural network are already impaired in non-metastatic non-small cell lung cancer (NSCLC) patients prior to chemotherapy, characterized by a reduced global clustering coefficient and an increased shortest path length in specific emotion-related brain regions (e.g., the hippocampus and prefrontal lobe) (17). This indicates that LC may disrupt the information integration and transmission efficiency of the white matter network through its paraneoplastic effect, offering an explanation for the neural basis of cancer-related emotional distress (e.g., depression and anxiety) in these patients. More importantly, abnormal white matter network function is directly associated with cognitive-emotional deficits. Studies have shown that the global and local efficiency of the frontal-subcortical white matter network is significantly reduced in NSCLC patients before chemotherapy, and this reduction correlates significantly with scores for memory impairment, inattention, and anxiety-depression (7). This directly confirms that decreased functional efficiency of the white matter network is the core neural mechanism underlying cognitive-emotional disorders in LC patients. To summarize, as the primary region housing the brain’s nerve fiber tracts, cerebral white matter structural integrity directly dictates the efficiency of neural signal transmission. As a key structure for maintaining central nervous system homeostasis, abnormal BBB permeability may represent the core mechanism by which LC’s paraneoplastic effect induces cerebral white matter microenvironmental dysregulation. Although existing studies—using diffusion tensor imaging (DTI)—have found that non-metastatic LC patients exhibit reduced white matter network topological efficiency (a change associated with cognitive-emotional impairments), and other studies—via texture analysis—have confirmed early microstructural changes in the bilateral frontoparietal white matter, these investigations have primarily focused on white matter fiber bundle integrity or macroscopic structural damage, with no in-depth exploration of microscopic pathophysiological alterations (e.g., BBB permeability). Additionally, the degree of abnormal white matter texture correlates with tumor stage and histopathological malignancy, suggesting that different LC pathological subtypes may affect the white matter microenvironment through distinct mechanisms. However, existing research has not clarified whether abnormal BBB permeability is involved in this process, nor has it examined regional specificity across distinct white matter areas (e.g., subcortical deep white matter and periventricular white matter). This limits a comprehensive understanding of the neural mechanisms underlying LC’s paraneoplastic effect and hinders the development of targeted brain metastasis risk stratification and neuroprotective strategies.

Based on the above analysis, this study hypothesizes that non-brain metastatic LC patients already exhibit abnormal BBB permeability in the cerebral white matter, which demonstrates regional and pathological subtype specificity. To verify this hypothesis, this study takes non-brain metastatic LC patients (with pathologically confirmed LC and no brain metastases on cranial MRI) as research objects, comparing them with two control groups: ① healthy controls (HC) with no abnormal findings on cranial MRI; ② LCBM patients (lung cancer with brain metastasis, LCBM) with pathologically confirmed LC and cranial MRI-detected tiny metastases (maximum diameter <0.5 cm). DCE-MRI is used to quantitatively analyze BBB permeability in the cerebral white matter (including frontal, parietal, temporal, occipital, and periventricular white matter), aiming to: (1) confirm the presence of abnormal BBB permeability in the cerebral white matter of non-brain metastatic LC patients; (2) explore the distribution differences of abnormal BBB permeability in different white matter regions; (3) provide imaging evidence for further investigating the mechanism of LC-related white matter injury and the pathophysiological basis of cognitive impairment and pain network dysfunction. By achieving the above goals, this study is expected to provide key theoretical support for the formulation of neuroprotective strategies in non-brain metastatic LC patients, thereby laying a foundation for optimizing brain metastasis risk stratification and individualized treatment regimens, with important theoretical value and clinical transformation significance.

## 2 Materials and methods

### 2.1 Patient characteristics

**Figure 1:**
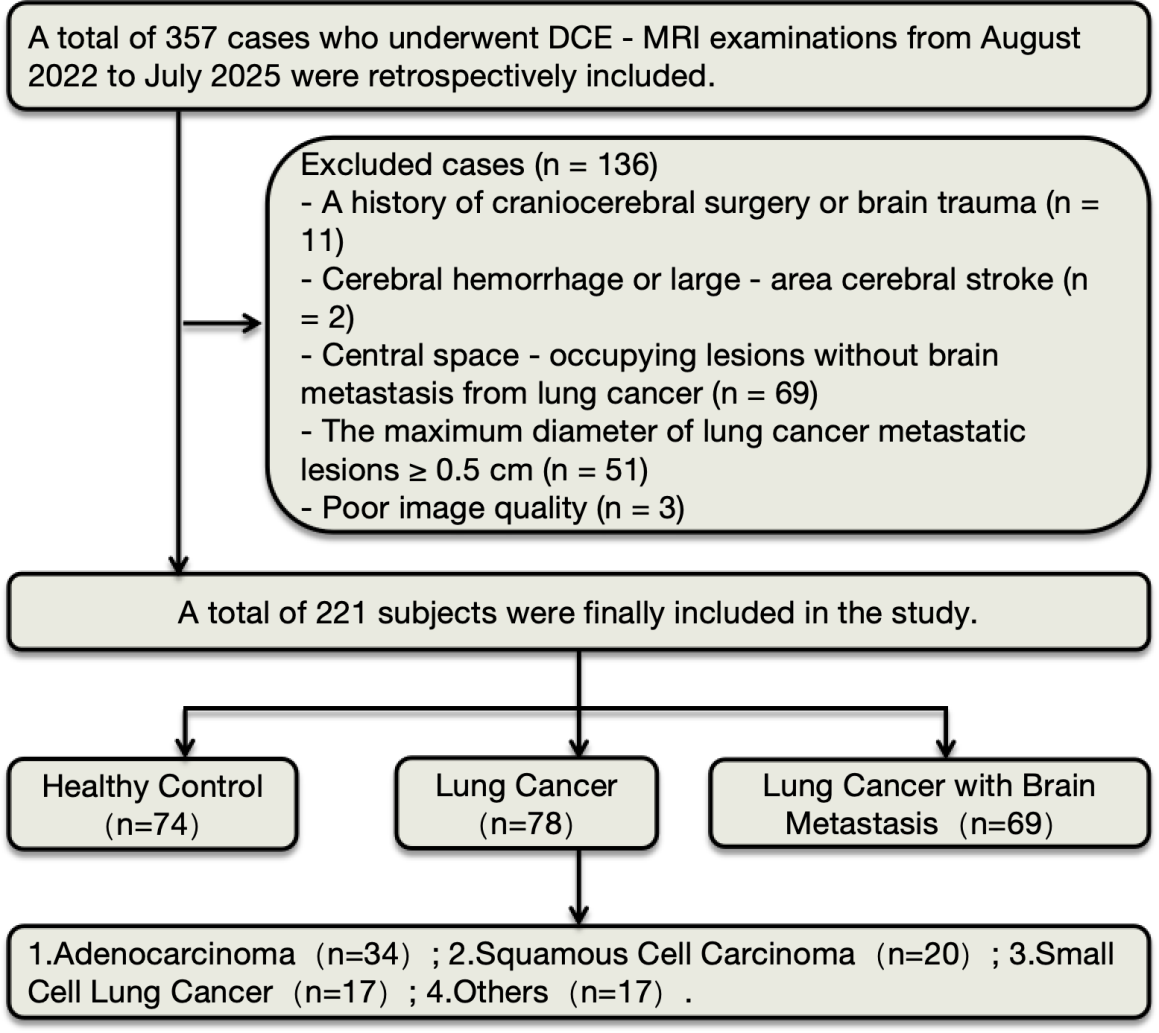
Study flowchart.

This study protocol was approved by the Ethics Committee of Jiangjin Hospital of Chongqing University (approval number: KY2023073) and strictly adhered to the principles of the Declaration of Helsinki, and the requirement for informed consent was waived. Overall, this study retrospectively evaluated 357 patients with suspected central nervous system (CNS) diseases who underwent DCE-MRI examination in the Radiology Department of our hospital from August 2022 to July 2025. Exclusion criteria were as follows: (i) History of previous craniocerebral surgery or traumatic brain injury; (ii) Cerebral hemorrhage or large-area cerebral infarction; (iii) Central nervous system space-occupying lesions that were not brain metastases from lung cancer; (iv) Metastatic lesions with maximum diameter ≥ 0.5 cm (with obvious mass effect); (v) Poor image quality of DCE-MRI (e.g., severe motion artifacts, failure of contrast agent injection, etc.). Finally, 221 patients were included in this study. See **Figure 1** for details of the inclusion and exclusion criteria.

### 2.2 Grouping criteria

Based on disease diagnosis results and brain metastasis status, all enrolled patients were divided into three groups:(i) HC: Subjects in this group underwent cranial MRI examination, which showed no abnormal intracranial findings.(ii) LC: All subjects were definitely diagnosed with lung cancer by pathological examination, and no intracranial brain metastatic lesions were detected by cranial MRI examination.(iii) LCBM): Subjects must meet two core criteria simultaneously: confirmed as LC by pathological examination, and definitely detected with intracranial brain metastatic lesions by cranial MRI examination. The maximum diameter of all metastatic lesions was less than 0.5 cm (such lesions typically have no obvious mass effect).

To further clarify the association between different histopathological types of lung cancer and cerebral white matterBBB, this study further subdivided LC patients. Given that the number of cases with three pathological types—adenocarcinoma (ADC), squamous cell carcinoma (SCC), and small cell lung cancer (SCLC)—was relatively large (with good statistical representativeness), this group was further divided into ADC subgroup, SCC subgroup, SCLC subgroup, and others for targeted analysis. The detailed patient grouping process is shown in **Figure 1**.

### 2.3 Examination methods

Imaging was performed using a United Imaging 3.0 T MRI scanner (model: UMR780). First, routine sequence scanning was conducted, followed by DCE-MRI sequence scanning. The DCE-MRI sequence consisted of two components: a multi-flip-angle scanning sequence with 5 flip angles and a subsequent multi-phase dynamic contrast-enhanced sequence. The multi-flip-angle scanning sequence included 5 single-phase flip-angle scans, with flip angles set in sequence as 3°, 6°, 9°, 12°, and 15°. Its imaging parameters were as follows: matrix = 112×100; field of view (FOV) = 230 mm × 200 mm; time of repetition (TR)/time of echo (TE) = 4.11 ms/1.84 ms; number of signal excitations = 1; number of slices = 20; slice thickness = 6 mm; slice gap = 0 mm; and slice direction difference = 2.For the multi-phase dynamic contrast-enhanced sequence, the flip angle was set to 10°, with TR/TE = 2.51 ms/0.92 ms and temporal resolution = 3 s. Other parameters (including number of slices, slice thickness, slice gap, slice direction difference, matrix, and FOV) were consistent with those of the single-phase flip-angle scanning sequence. A total of 90 phases were scanned, generating 3,600 images, with the total scanning duration for multi-phase dynamic perfusion being 253 seconds. Contrast agent administration was performed at the end of the 5th phase and the start of the 6th phase of the multi-phase dynamic sequence: gadobutrol was injected via the elbow vein using a high-pressure syringe at a rate of 3 mL/s, followed by 20 mL of 0.9% sodium chloride solution administered at the same rate after completion of contrast agent injection.

### 2.4 Image post-processing

**Figure 2:**
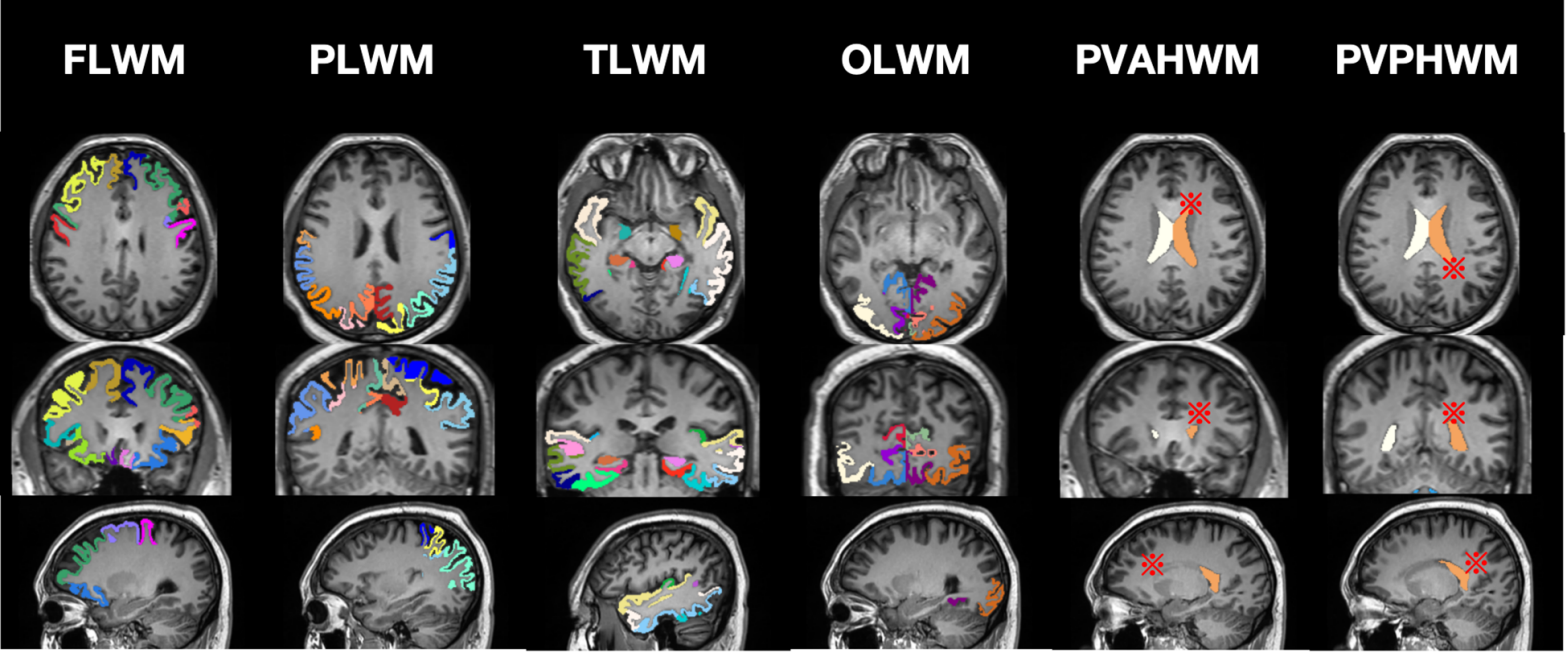
Schematic diagram of ROI delineation in each region. The colored regions in the figure represent the Cerebral Cortex, which serves to assist in localizing ROI delineation for white matter regions. FLWM, Frontal Lobe White Matter; PLWM, Parietal Lobe White Matter; TLWM, Temporal Lobe White Matter; OLWM, Occipital Lobe White Matter; PVAHWM, Periventricular Anterior Horn White Matter; PVPHWM, Periventricular Posterior Horn White Matter. ※Schematic representation of periventricular white matter delineation regions.

The Extended Tofts two-compartment model was used for post-processing of DCE-MRI data in this study: First, image motion correction was performed to eliminate motion artifacts; the middle cerebral artery was selected as the arterial input function and the superior sagittal sinus as the venous output function to obtain time-signal intensity curves; regions of interest (ROI) were manually delineated on slices where gray and white matter were clearly displayed in the T1WI-Flair sequence, with an area greater than 150 mm². Representative regions were selected according to anatomical distribution, including frontal lobe white matter, parietal lobe white matter, temporal lobe white matter, occipital lobe white matter, white matter of the anterior horn of the lateral ventricle, and white matter of the posterior horn of the lateral ventricle. ROIs were delineated on both left and right sides, and the mean value was taken, avoiding cerebral sulci and ventricles (see **Figure 2**).

### 2.5 Statistical analysis

GraphPad Prism 9 was employed for data processing and graph generation in this study. For quantitative data, normality was first assessed using the Kolmogorov-Smirnov test: Data with a normal distribution were expressed as mean ± standard deviation, and comparisons between two groups were performed using the independent samples LSD-t test. Non-normally distributed data were expressed as median (interquartile range) [M (P25, P75)], and comparisons between two groups were performed using the Mann-Whitney U test. For correlation analysis, the Pearson correlation coefficient was used for normally distributed data, while Spearman’s rank correlation coefficient was used for non-normally distributed data. All statistical tests were two-tailed, and P value < 0.05 was considered statistically significant.

## 3 Results

### 3.1 Baseline characteristics of the patients

Detailed information on demographic characteristics (age, gender) of the subjects and pathological subtypes of lung cancer is provided in **Table 1**.

**Table 1:** Demographic characteristics of each group.

Parameters	HC(n=74)	LC(n=78)	LCBM(n=69)
Age (Mean ± Variance)	64.80 ± 12.79	65.54 ± 9.11	64.65 ± 8.45
Gender(n, %)			
Male	34(45.95%)	63(80.77%)	48(69.57%)
Female	40(54.05%)	15(19.23%)	21(30.43%)
Histopathology(n, %)			
Adenocarcinoma	NA	34(43.59%)	41(59.42%)
Squamous Cell Carcinoma	NA	20(25.64%)	10(14.49%)
Large Cell Lung Cancer	NA	1(1.28%)	0(0.00%)
Small Cell Lung Cancer	NA	17(21.79%)	8(11.59%)
Mucoepidermoid Carcinoma	NA	2(2.56%)	0(0.00%)
Sarcomatoid Carcinoma	NA	0(0.00%)	1(1.45%)
Unknown Pathology	NA	4(5.13%)	9(13.04%)

HC, Healthy Control; LC, Lung Cancer; LCBM, Lung Cancer with Brain Metastasis; NA, Not Applicable.

### 3.2 Analysis of BBB permeability differences in different cerebral white matter regions among HC, LC, and LCBM

**Figure 3:**
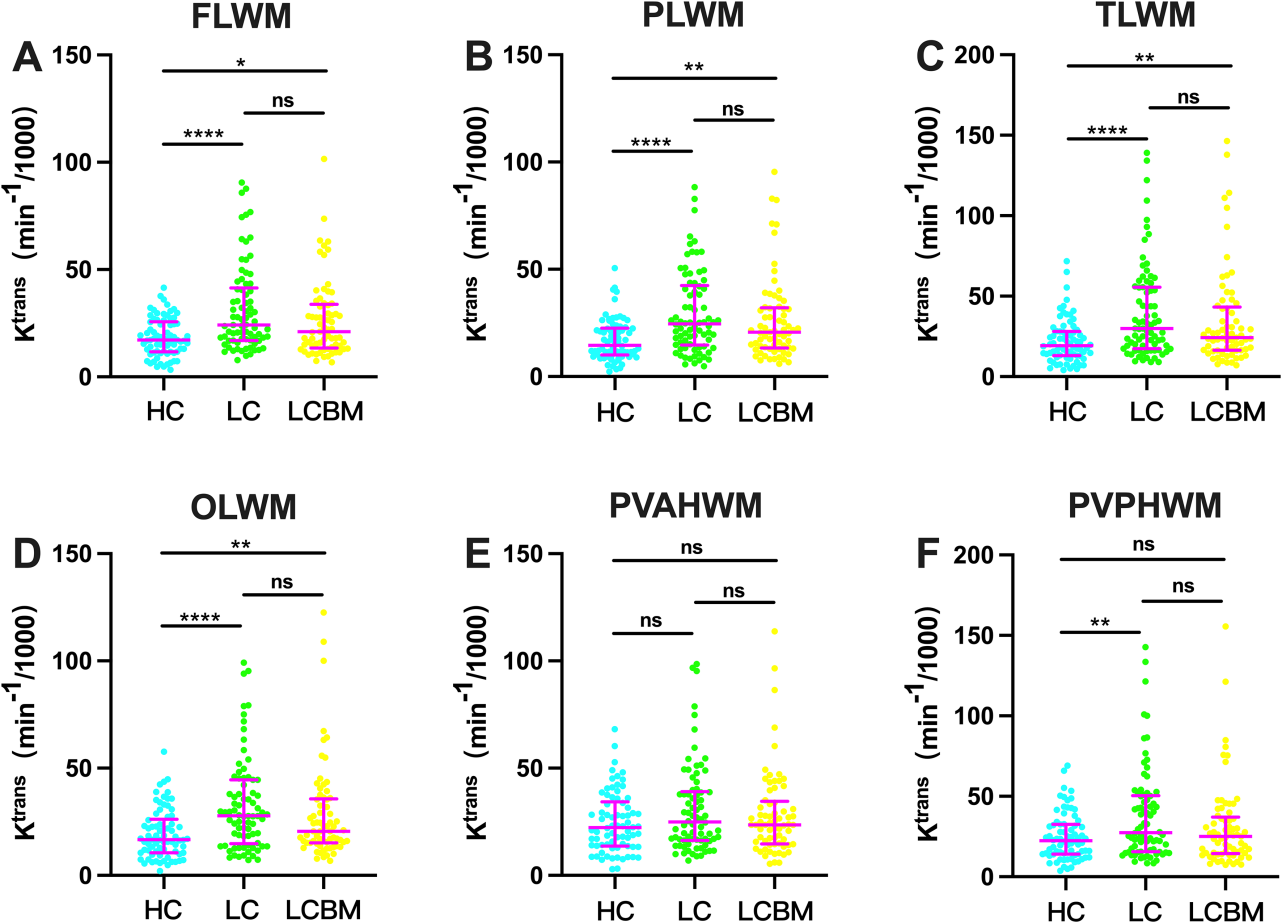
BBB permeability differences in cerebral white matter regions among lung cancer, healthy control, and brain metastasis groups. HC, Healthy Control; LC, Lung Cancer; LCBM, Lung Cancer with Brain Metastasis; FLWM, Frontal Lobe White Matter; PLWM, Parietal Lobe White Matter; TLWM, Temporal Lobe White Matter; OLWM, Occipital Lobe White Matter; PVAHWM, Periventricular Anterior Horn White Matter; PVPHWM, Periventricular Posterior Horn White Matter. *p<0.05; **p<0.01; ****p<0.0001; ns indicates no statistical significance.

As shown in **Figure 3**, the k^trans^ in different cerebral white matter regions exhibited characteristic differences among the HC, LC, and LCBM groups. Specifically, no statistically significant differences in k^trans^ values were observed between the LC and LCBM groups across all cerebral white matter regions, including the frontal lobe, parietal lobe, temporal lobe, occipital lobe, anterior periventricular horn, and posterior periventricular horn (all p>0.05). In comparison with the HC group, the LC group showed significantly increased k^trans^ values in the frontal lobe, parietal lobe, temporal lobe, occipital lobe, and posterior periventricular horn white matter regions (all p<0.05), whereas no statistically significant difference was noted in the anterior periventricular horn white matter region (p>0.05). Meanwhile, the LCBM group displayed significantly higher k^trans^ values than the HC group in the frontal lobe, parietal lobe, temporal lobe, and occipital lobe white matter regions (all p<0.05); however, no significant differences were found between the two groups in the periventricular white matter regions (p>0.05).

### 3.3 Analysis of differences in BBB permeability in different cerebral white matter regions among different histopathological types of LC

**Figure 4:**
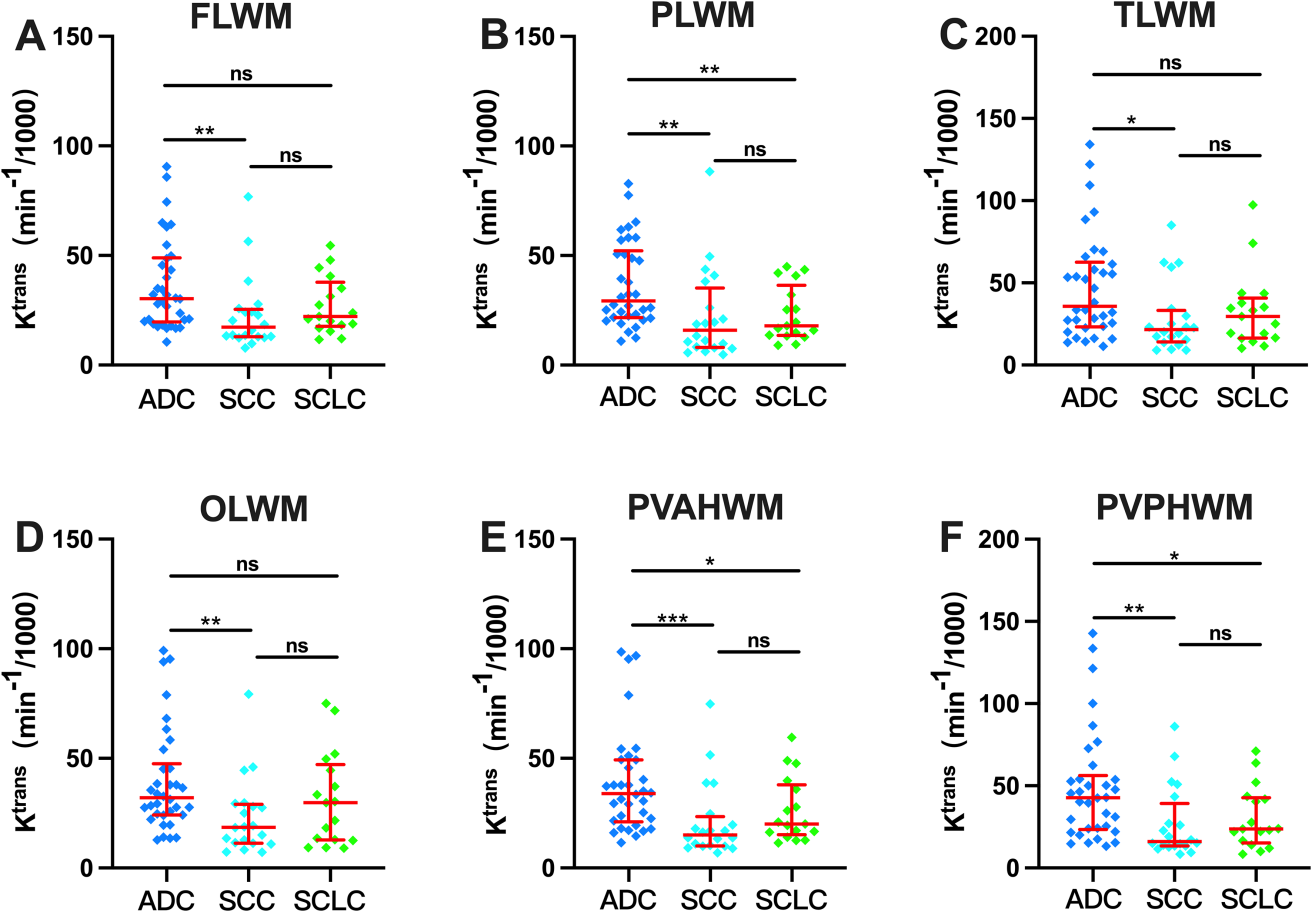
BBB permeability differences in white matter regions of lung cancer patients with different histopathological types. ADC, Adenocarcinoma; SCC, Squamous Cell Carcinoma; SCLC, Small Cell Lung Cancer; FLWM, Frontal Lobe White Matter; PLWM, Parietal Lobe White Matter; TLWM, Temporal Lobe White Matter; OLWM, Occipital Lobe White Matter; PVAHWM, Periventricular Anterior Horn White Matter; PVPHWM, Periventricular Posterior Horn White Matter. *p<0.05; **p<0.01; ***p<0.001; ****p<0.0001; ns indicates no statistical significance.

This section focuses on the three most common clinical histopathological types (Adenocarcinoma [ADC], Squamous Cell Carcinoma [SCC], and Small Cell Lung Cancer [SCLC]) to investigate differences in the impact of different histopathological types of LC on BBB permeability in cerebral white matter. As shown in **Figure 4**, compared with the SCC group, the ADC group exhibited significantly higher k^trans^ values in all cerebral white matter regions, including the frontal lobe, parietal lobe, temporal lobe, occipital lobe, anterior periventricular horn, and posterior periventricular horn (all p<0.05). Furthermore, compared with the SCLC group, the ADC group also showed significantly increased k^trans^ values in the parietal lobe, anterior periventricular horn, and posterior periventricular horn white matter regions (p<0.05). Whereas, the SCC group and the SCLC group exhibited no statistically significant differences in k^trans^ values across all cerebral white matter regions (p>0.05).

### 3.4 Differences in cerebral white matter DCE-MRI quantitative parameters among lung cancer patients with different histopathological subtypes

DCE-MRI quantitative parameters were analyzed by combining all cerebral white matter regions (frontal lobe, parietal lobe, temporal lobe, occipital lobe, anterior periventricular horn, and posterior periventricular horn). As shown in **Table 2**, differences in cerebral white matter DCE-MRI quantitative parameters were observed among lung cancer patients with different histopathological subtypes: For the K^trans^, the median value in the ADC group was 33 (22.04, 53.79) min^-1^/1000, which was significantly higher than that in the SCC group [17.33 (11.73, 27.74) min^-1^/1000, p<0.0001] and the SCLC group [22.68 (15.30, 40.43) min^-1^/1000, p<0.0001].In terms of the K_ep_, the median value in the ADC group was 4189 (3145, 5485) min^-1^/1000, which was significantly higher than that in the SCC group [2970 (1976, 4137) min^-1^/1000, p<0.0001] and the SCLC group [2874 (1554, 3876) min^-1^/1000, p<0.0001].Regarding the Ve, the median value in the ADC group was 9.85 (6.56, 13.39)/1000, which was significantly higher than that in the SCC group [7.18 (5.40, 11.54)/1000, p=0.001] but significantly lower than that in the SCLC group [10.60 (7.79, 20.99)/1000, p=0.003].For the Vp, the median value in the ADC group was 5.80 (3.95, 8.36)/1000, which was significantly higher than that in the SCC group [4.40 (3.30, 6.66)/1000, p=0.002], with no significant difference compared to the SCLC group [5.75 (3.70, 9.61)/1000, p=0.71].

**Table 2:** Compares DCE MRI parameters across pathological subtypes in lung cancer.

Parameters	ADC(n=34, ROI = 204)	SCC(n=20, ROI = 120)	SCLC(n=17, ROI = 102)	U	*p*
K^trans^(min^-1^/1000)	33(22.04, 53.79)	17.33(11.73, 27.74)	22.68(15.30, 40.43)	6057^$^,6981^&^, 4749^¥^	<0.0001^$^,<0.0001^&^,0.004^¥^
K_ep_(min^-1^/1000)	4189(3145, 5485)	2970(1976, 4137)	2874(1554, 3876)	7980^$^,5591^&^, 5397^¥^	<0.0001^$^,<0.0001^&^, 0.13^¥^
Ve(1/1000)	9.85(6.56, 13.39)	7.18(5.40, 11.54)	10.60(7.79, 20.99)	9626^$^,8206^&^,3577^¥^	0.001^$^,0.003^&^, <0.0001^¥^
Vp(1/1000)	5.80(3.95, 8.36)	4.40(3.30, 6.66)	5.75(3.70, 9.61)	9649^$^,10135^&^, 4864^¥^	0.002^$^,0.71^&^, 0.008^¥^

^$^Adenocarcinoma vs. Squamous Cell Carcinoma; ^&^Adenocarcinoma vs. Small Cell Lung Cancer; ^¥^Squamous Cell Carcinoma vs. Small Cell Lung Cancer.

### 3.5 Correlation analysis between K^trans^ and Vp in cerebral white matter of patients with different histopathological types of lung cancer

**Figure 5:**
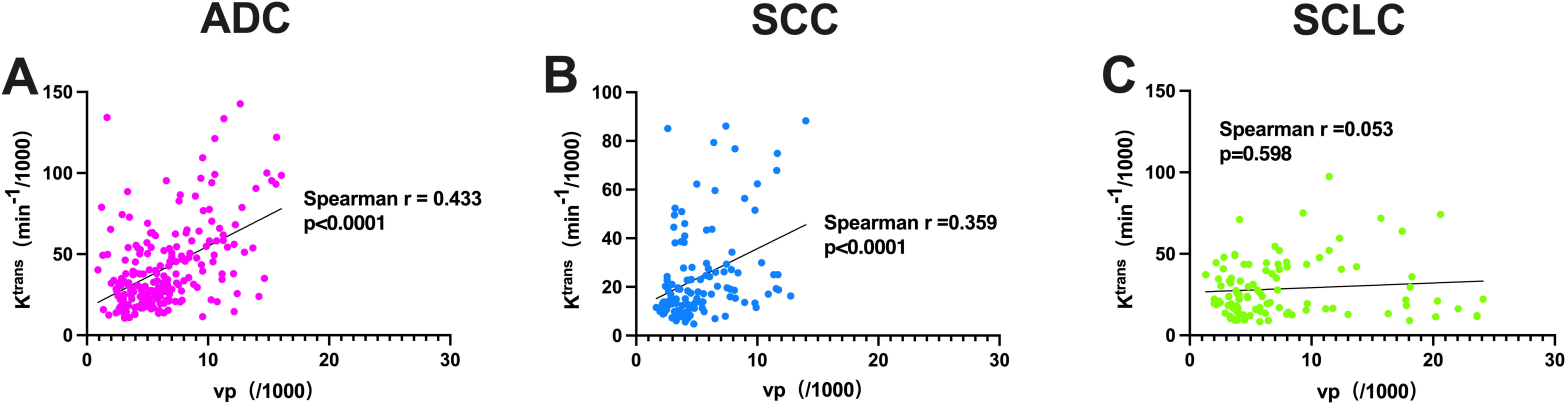
Correlation of Ktrans and Vp in cerebral white matter of lung cancer patients with different histopathological types. ADC, Adenocarcinoma; SCC, Squamous Cell Carcinoma; SCLC, Small Cell Lung Cancer.

As shown in **Figure 5**, Spearman’s rank correlation coefficient was used to analyze the correlation between K^trans^ and Vp in cerebral white matter of patients with different histopathological types of lung cancer, and the results showed that: In the ADC group, K^trans^ and Vp in cerebral white matter showed a significant positive correlation (r=0.433, p<0.0001); In the SCC group, K^trans^ and Vp in cerebral white matter also showed a significant positive correlation (r=0.359, p<0.0001); Whereas in the SCLC group, there was no significant correlation between K^trans^ and Vp in cerebral white matter (r=0.053, p=0.598).

## 4 Discussion

This study aimed to investigate whether there are abnormalities in the BBB permeability across different white matter regions in LC patients without brain metastasis, and to explore the correlation between such abnormalities and the histopathology of LC. Quantitative analysis using DCE-MRI confirmed that LC patients without brain metastasis exhibited a significant and widespread increase in BBB permeability in cerebral white matter. This finding suggests that LC can impair the structural integrity of the BBB in cerebral white matter through paraneoplastic effects even before the occurrence of brain metastasis. Further analysis revealed significant heterogeneity in cerebral white matter BBB permeability among LC patients with different histopathological subtypes: ADC was characterized by high perfusion and prominent vascular leakage, whereas SCLC was primarily associated with interstitial damage. In terms of regional distribution, the increased BBB permeability in ADC patients involved the entire cerebral white matter, while in SCLC patients, the impairment was mainly confined to the deep white matter regions. These findings provide crucial imaging evidence for risk stratification of brain metastasis in LC patients and the formulation of individualized neuroprotective strategies.

Through quantitative DCE-MRI analysis, this study establishes that the LC cohort exhibits pervasive BBB permeability abnormalities in the cerebral white matter prior to overt brain metastasis. Specifically, relative to the HC group, the LC cohort demonstrated significantly elevated K^trans^ values in the frontal, parietal, temporal, and occipital lobe white matter regions, as well as the posterior periventricular horn (all p<0.05). Moreover, the magnitude of permeability elevation in the LC group did not differ significantly from that observed in LCBM patients with metastatic lesions <0.5 cm in diameter (p>0.05), indicating that LC induces cerebral white matter BBB disruption via paraneoplastic effects before the onset of detectable brain metastases. Stratified subgroup analysis further delineated that abnormal white matter permeability in the LC cohort is characterized by histopathological specificity and distinct regional distribution patterns. The ADC subgroup showed significantly greater K^trans^, K_ep_, and Vp values compared with both the SCC and SCLC subgroups (all p<0.001), with a robust positive correlation between Vp and K^trans^ in white matter regions (r=0.433, p<0.001). This indicates that pronounced BBB leakage in the ADC subgroup is predominantly driven by hyperperfusion status and enhanced microvascular permeability. In contrast, the SCLC subgroup exhibited significantly elevated Ve values relative to the ADC subgroup (p=0.003), with no significant correlation between Vp and K^trans^ observed in its white matter regions (r=0.053, p=0.598). This implies that the heightened BBB permeability in the SCLC subgroup is centered on interstitial structural disruption. The SCC subgroup, marked by low angiogenic potential and a muted inflammatory response, displayed the lowest BBB permeability across the three subtypes. Collectively, these findings delineate three distinct paraneoplastic effect profiles in lung cancer, each linked to unique pathophysiological mechanisms. Furthermore, no statistically significant difference in BBB permeability was observed between the SCLC and ADC subgroups in deep white matter regions (frontal, temporal, and occipital lobes; all p>0.05). This suggests that SCLC-specific disruption of deep white matter structures narrows the disparity in BBB leakage between SCLC and ADC subgroups within these regions. This subtype-specific classification, derived from DCE-MRI parameters, addresses a critical research gap in elucidating the imaging mechanisms underlying cerebral white matter injury in non-brain metastatic LC. It also furnishes essential imaging evidence to support the development of personalized neuroprotective strategies, thereby advancing precision oncology for lung cancer patients at risk of neurological complications.

The core conclusions of this study—namely, that non-brain metastatic LC disrupts the structural integrity of the cerebral white matter BBB through “paraneoplastic effects” and that distinct histopathological subtypes of LC induce differential alterations in the cerebral white matter microenvironment—are highly consistent with findings from most previous studies regarding LC brain metastasis mechanisms, white matter injury, and pathological subtype specificity. Additionally, this study provides novel evidence for evaluating the pre-metastatic cerebral white matter microenvironment. Wei et al. (18) confirmed via a large-scale retrospective study that the proportion of SCLC brain metastases located in deep white matter (22.51%) was significantly higher than that of NSCLC (17.96%). The anatomical basis for this may be attributed to abrupt reductions in vascular caliber and hemodynamic changes in these regions, which facilitate the capture of circulating tumor cells, ultimately leading to the preferential distribution of SCLC metastases. Further analysis in the present study revealed that, even at the non-brain metastatic stage, SCLC already alters the deep white matter microenvironment through paraneoplastic effects, characterized by a significant increase in the Ve. Notably, BBB permeability in deep white matter regions (e.g., frontal, parietal, and temporal lobes) did not differ significantly between the SCLC group and the ADC group (which exhibited marked BBB leakage) (p>0.05). This suggests that SCLC-induced specific disruption of deep white matter structures narrows the gap in BBB leakage between SCLC and ADC groups in these regions, a finding that corroborates the hypothesis proposed by Yong et al. (19) that “SCLC promotes metastasis through microenvironmental remodeling.” It implies that SCLC may create a “pre-metastatic niche” for subsequent metastatic colonization by expanding the extracellular space prior to metastasis. In terms of molecular mechanisms, Bihong et al. (20) noted that the spatial distribution of brain metastases from ALK+ NSCLC differs from other mutation types, often involving white matter regions such as the right middle occipital gyrus and posterior cingulate gyrus, suggesting that driver gene mutations may influence metastatic localization by regulating organotropism of tumor cells. Tia et al. (21) further confirmed that ALK inhibitor treatment alters metastatic characteristics: ALK+ patients not receiving tyrosine kinase inhibitors (TKIs) develop larger brain metastases, whereas those treated with crizotinib are more prone to metastases entirely confined to white matter. This suggests that poor central nervous system distribution of drugs in white matter regions may contribute to a “spatially mediated resistance mechanism.” Although the present study did not directly investigate genetic mutations, it found that ADC (the main subtype of NSCLC) drives global cerebral white matter leakage through “enhanced vascular permeability,” whereas SCLC preferentially affects deep white matter through “extracellular space remodeling,” providing imaging evidence for the association between molecular subtypes and BBB injury mechanisms. Regarding the association between BBB injury and neurological function, Zhang et al. (22) demonstrated via cerebrospinal fluid analysis that BBB leakage (albumin index) in non-brain metastatic LC patients correlates significantly with impaired executive function. The present study further reveals that this association exhibits pathological subtype specificity: the high K^trans^ values (33 min^-1^/1000) in the ADC group are more strongly correlated with reduced frontal-subcortical network efficiency (7), whereas the high Ve values in the SCLC group may impair neural signal transmission through extracellular space expansion. The causal metabolic covariance network (CaMCN) approach proposed by Tianzheng et al. (23) further revealed that NSCLC can affect glucose uptake at the gray-white matter junctions of the frontal and temporal lobes through “paraneoplastic effects.” Yu et al. (24) also identified abnormal metabolic network connectivity in the frontal and temporal lobes of NSCLC patients using PET imaging, which aligns with the regional distribution of abnormal BBB permeability in deep white matter observed in this study. These findings suggest that metabolic microenvironmental changes may synergize with BBB injury to contribute to the pathological process of cognitive impairment. Notably, Simó et al. (8) identified reduced fractional anisotropy (FA) of white matter fiber bundles in lung cancer patients after chemotherapy using diffusion tensor imaging (DTI). In contrast, the present study observed abnormal BBB permeability in non-brain metastatic LC patients, suggesting that the impact of LC itself on cerebral white matter may be independent of therapeutic interventions. This is consistent with Takeshita et al.’s (10) conclusion that “radiotherapy has limited direct damage to cerebral white matter,” collectively supporting the view that “LC paraneoplastic effects are an early driver of white matter abnormalities.” Furthermore, Li et al. (13) used automatic fiber quantification (AFQ) to demonstrate reduced FA in the right cingulum-hippocampus of LC patients with cancer pain, with FA values negatively correlating with Numeric Rating Scale (NRS) scores for pain. Yu et al. (11) further identified gray matter volume reductions in regions such as the medial frontal gyrus (MFG) and right middle temporal gyrus in lung cancer patients with bone metastatic pain using voxel-based morphometry (VBM), along with enhanced functional connectivity between the MFG and bilateral cerebellar lobule VI. These structural and functional network abnormalities in gray matter, together with BBB permeability anomalies observed in this study, constitute the neural basis of lung cancer-related cognitive-pain comorbidity. Xu et al. (16) confirmed via MRI texture analysis that significant abnormal texture features already exist in the bilateral frontoparietal white matter of LC patients without brain metastasis, with more pronounced abnormalities in the SCLC group compared to the ADC group, suggesting that LC may induce microstructural changes in cerebral white matter through paraneoplastic mechanisms such as cytokine secretion. This study complements the BBB permeability findings of the present study from the perspective of quantitative tissue microstructure analysis, collectively supporting the view that “LC disrupts the cerebral white matter microenvironment through paraneoplastic effects prior to brain metastasis. “A key difference from existing studies lies in the BBB characteristics of the SCC group. The present study found that K^trans^ values in the SCC group (17.33 min^-1^/1000) were significantly lower than those in the ADC and SCLC groups and close to those in healthy controls, consistent with the clinical observation of a lower brain metastasis rate in the SCC group (14.49%, **Table 1**). This suggests that BBB permeability is not the sole driver of cognitive impairment and highlights the need for comprehensive assessment combining white matter microstructure and functional networks. In summary, building on the validation of existing conclusions—such as SCLC’s preference for deep white matter and the association between BBB injury and cognition—this study is the first to reveal differential mechanisms of distinct pathological subtypes at the non-brain metastatic stage, filling the research gap in “pre-metastatic microenvironmental changes” and providing a novel perspective for understanding the spatiotemporal progression of lung cancer-related cerebral white matter injury.

The findings of this study hold significant clinical translational value and provide critical imaging evidence for the early warning and intervention of LC brain metastasis. In terms of clinical significance, abnormal BBB permeability in the cerebral white matter of patients with non-brain metastatic LC—particularly high K^trans^ in ADC and high Ve in SCLC—may serve as potential biomarkers for risk stratification of brain metastasis. For ADC patients, a significant increase in deep white matter K^trans^ values (median 33 min^-1^/1000) indicates enhanced vascular permeability, warranting priority consideration of VEGF/MMPs inhibitors (e.g., bevacizumab) combined with BBB protectants (e.g., edaravone) to reduce metastasis risk. Notably, the third-generation EGFR-TKI osimertinib has been confirmed to possess favorable BBB penetration, with brain exposure levels in healthy subjects comparable to central nervous system drugs (25) and demonstrated significant efficacy in EGFR-mutant positive NSCLC brain metastasis models. Future studies could build on the identified BBB permeability abnormalities to explore individualized regimens combining BBB protectants and targeted agents for high-risk patients (e.g., ADC) to further reduce brain metastasis risk. For SCLC patients, even in the absence of significant K^trans^ elevation, high Ve values in deep white matter (10.60/1000) still necessitate vigilance for deep white matter metastasis in conjunction with prophylactic cranial irradiation (PCI). This complements the conclusion by Rob et al. (26) that “hippocampal-avoidance PCI preserves white matter networks,” providing a basis for formulating individualized radiotherapy regimens. This study reveals important insights for clinical practice: BBB permeability in LCBM patients was not significantly higher than in LC patients; in fact, it was even lower in some critical brain regions (e.g., deep white matter, periventricular white matter). A potential explanation is that metastatic lesions sequester local blood flow via a “steal effect,” reducing perfusion and contrast agent leakage in surrounding normal white matter, leading to a “pseudo-decrease” in DCE-MRI parameters such as K^trans^. Clinically, therefore, dynamic contrast-enhanced MRI monitoring of hemodynamic associations between metastatic lesions and surrounding white matter is necessary to avoid underestimating potential metastasis risk by relying solely on K^trans^ values. At the diagnostic technology level, conventional MRI plain scan struggles to identify pre-metastatic BBB microenvironmental abnormalities, whereas the quantitative DCE-MRI parameters (K^trans^, Ve) used in this study can sensitively capture early changes. For example, abnormal K^trans^ in periventricular white matter of SCLC patients precedes detectable metastases on structural MRI, providing a “window period” for clinical intervention. It is recommended that high-risk populations (e.g., ADC, SCLC) undergo routine DCE-MRI examinations, with warning thresholds set at a volume transfer constant K^trans^ >22 min^-1^/1000 or extracellular volume fraction Ve >10/1000.More importantly, this study confirms “subtype-regional” specificity in the impact of LC on cerebral white matter BBB: ADC drives global white matter leakage via “enhanced vascular permeability,” SCLC is characterized by “extracellular space remodeling” with preferential involvement of deep white matter, while SCC exhibits low BBB permeability across all brain regions due to low-inflammatory and low-angiogenic phenotypes. This finding advances monitoring strategies toward “subtype individualization”—for instance, ADC patients could undergo repeated DCE-MRI every 3 months to track K^trans^ changes, while SCLC patients require simultaneous attention to Ve values and associations with cerebrospinal fluid myelin damage markers (e.g., neurofilament light chain protein), enabling a shift from “pre-metastatic intervention” to “subtype-specific prevention. “In summary, through quantitative DCE-MRI technology, this study deepens the understanding of LC-induced cerebral white matter BBB abnormalities from “macroscopic phenomena” to “subtype-specific mechanisms,” providing actionable risk stratification tools for clinical practice and laying an imaging foundation for precise prevention and treatment of LC neurological complications.

This study has certain practical limitations, which provide important references for future research directions but do not affect the reliability of the core conclusions. First, the retrospective nature of the study design may introduce selection bias. The included cases were all from a single center, and confounding factors such as age and underlying diseases were not strictly matched, which may have a potential impact on intergroup comparisons of cerebral white matter BBB parameters. Second, due to the sample size limitation in pathological subtype analysis, rare subtypes such as large cell lung cancer were not included in the statistics due to insufficient case numbers, failing to fully reflect the BBB characteristics of all LC pathological types. Additionally, further analysis of the dynamic effects of tumor stage, treatment history (e.g., chemotherapy, targeted therapy), and disease duration on BBB permeability was not performed. These factors may indirectly regulate BBB integrity through inflammatory responses or changes in the vascular microenvironment (27–29).Furthermore, regarding the indirectness of mechanism exploration, this study inferred potential mechanisms of lung cancer paraneoplastic effects (e.g., VEGF-mediated enhancement of vascular permeability, expansion of extracellular space caused by neuroinflammation) through DCE-MRI parameters. However, direct evidence such as cerebrospinal fluid inflammatory factor detection and pathological biopsy is lacking, making it impossible to clarify the molecular cascade reactions of BBB damage. Finally, due to the lack of association with clinical symptoms, correlation analysis between BBB parameters and clinical indicators such as cognitive function scores (e.g., MoCA, MMSE) and visual analog scale (VAS) for cancer pain was not performed, making it difficult to directly explain the actual impact of BBB abnormalities on patients’ neural function. Quantitative analysis via DCE-MRI confirmed that patients with LC exhibit abnormally increased BBB permeability in cerebral white matter, and this abnormality presents distinct regional specificity and pathological subtype-specific characteristics: ADC is characterized by high perfusion and increased vascular leakage, involving global cerebral white matter; SCLC is centered on interstitial structural disruption, primarily affecting deep white matter regions; SCC, due to its low angiogenic potential and weak inflammatory response, exhibits the lowest BBB permeability. This study reveals the dual “subtype-regional” specific mechanism underlying the impact of LC on cerebral white matter BBB during the non-brain metastatic stage, filling the research gap in “pre-metastatic cerebral white matter microenvironmental changes” and providing an imaging perspective for understanding the pathophysiological basis of LC-related neurological complications. Additionally, through quantitative DCE-MRI parameters, potential biomarkers applicable for brain metastasis risk stratification have been established, offering critical imaging evidence for the clinical development of individualized neuroprotective strategies and promoting the shift of LC brain metastasis management from “passive treatment” to “active prevention”.

## Data Availability

The raw data supporting the conclusions of this article will be made available by the authors, without undue reservation.
